# Optimal exercise temporal parameters of Traditional Chinese Exercises for cognitive function of older adults with mild cognitive impairment: a systematic review and dose–response meta-analysis of randomized controlled trials

**DOI:** 10.3389/fmed.2025.1568835

**Published:** 2025-05-07

**Authors:** Qingpan Wen, Qin Luo, Yangjun Liu, Lu Li, Marcin Białas, Dominika Wilczyńska

**Affiliations:** ^1^Faculty of Physical Education, Gdansk University of Physical Education and Sport, Gdańsk, Poland; ^2^Department of Rheumatology and Orthopedics, Sichuan Province Orthopedic Hospital, Chengdu, China; ^3^Faculty of Social and Humanities, WSB Merito University Gdansk, Gdańsk, Poland

**Keywords:** exercise therapy, cognitive functions, exercise parameters, mild cognitive impairment, Traditional Chinese Exercises

## Abstract

**Background:**

Mild cognitive impairment (MCI) is characterized by a progressive decline in memory and other cognitive functions, falling between normal cognition and dementia. Traditional Chinese Exercises (TCEs) have been proven effective for managing MCI. A dose–response meta-analysis was conducted to evaluate the correlation between exercise temporal parameters and their effectiveness in older adults with MCI.

**Methods:**

Randomized controlled trials (RCTs) on TCEs for MCI were searched across eight databases from their inception to September 2024. Literature was screened based on inclusion and exclusion criteria. Data from the selected studies were extracted, and the risk of bias was assessed using the RoB2 tool. The quality of the included studies was evaluated using the PEDro scale. The visualizations were conducted using the “robvis” package in R 4.3.3 software, while Stata 15.0 software was used to analyze the dose–response relations.

**Result:**

Out of 2,216 records,17 RCTs were included in the meta-analysis. Although significant heterogeneity was present among the studies, sensitivity analysis demonstrated good robustness. The results revealed significant improvements in cognitive function among older adults with MCI in the TCEs group: Montreal Cognitive Assessment (MoCA) (SMD = 1.04, 95% CI: 0.71–1.38) and Mini-Mental State Examination (MMSE) (SMD = 0.82, 95% CI: 0.41–1.42). The relations between exercise cycle, frequency, and overall cognitive function (MoCA and MMSE) followed a “*Λ*”-shaped curve. For MoCA, the relations with exercise duration also exhibited a “Λ”-shaped curve, while the relations between duration and MMSE was nonlinear. The peak improvements in MoCA and MMSE were observed at 12 weeks (25.59, 95% CI: 25.07–26.10) and 13 weeks (26.24, 95% CI: 25.38–27.09). Improvement was positively correlated with the number of cycles up to a peak, after which it declined, following a “*Λ*”-shaped pattern.

**Conclusion:**

This study demonstrates a nonlinear dose–response relations between exercise temporal parameters and therapeutic effects on cognitive function in older adults with MCI. Regarding exercise cycle, MoCA and MMES yield the optimal outcomes at 12 and 13 weeks. For exercise frequency, MoCA and MMES optimize results at three times per week. Concerning exercise duration, MoCA achieves optimal results at 45 min; MMES shows gradual improvement after 30 min.

**Systematic review registration:**

The study protocol was registered with PROSPER on May 29, 2024, under the registration number CRD42024510378, https://www.crd.york.ac.uk/PROSPERO/view/CRD42024510378

## 1 Introduction

More than 55 million people worldwide are currently living with dementia, a condition that is growing at an alarming rate. The number is projected to reach 78 million by 2030 and 115.4 million by 2050 (1, 2). Alzheimer’s disease (AD) is the most common cause of dementia, accounting for 60–80% of all cases (3, 4). Mild cognitive impairment (MCI) is often considered a transitional stage between normal cognitive function and clinically probable AD (5–7). While some older adults with MCI remain stable or even return to normal cognitive function over time, more than 50% progress to AD within 5 years (8). Active interventions during the MCI stage may effectively prevent or slow the progression to AD (9, 10). Engaging in regular physical exercise has been shown to alleviate the continuous decline in cognitive function and serves as a protective factor against the progression from MCI to dementia (11–13). Additionally, physical exercise has been proven to reduce the risk of cognitive impairment in older adults (14).

Traditional Chinese Exercises (TCEs) are comprehensive mind–body exercise approaches that involve the regulation of breathing, body movements, and consciousness as core components. The specific items included in TCEs, such as Tai Chi, Baduanjin, Wuqinxi, Yijinjing, and Liuzijue, etc. TCEs have been shown to improve cognitive function and provide long-term clinical benefits (15–17). Tai Chi exercise as well as Qigong enhances cognitive function and reduces the risk of falls (18). Baduanjin exercise improves cognitive function and attention (19, 20). Wuqinxi exercise may slow the deterioration of working memory (21), while Liuzijue exercise enhances memory and has a positive impact on brain health (22). These studies collectively demonstrate that TCEs are effective and viable methods for enhancing cognitive function. In clinical practice, therapeutic outcomes may vary depending on the frequency, duration, and cycles of TCEs. Some researchers recommend at least 12 weeks of practice, with sessions three times per week, lasting 30–60 min each (23). Others suggest sessions of 30–90 min, performed 3–6 times per week, over a total cycle of 8–36 weeks (24). Additionally, certain scholars propose a regimen of 3 months, with three sessions per week, each lasting 40 min (25). The aforementioned meta-analysis highlights significant differences in the recommended exercise parameters for TCEs. However, evidence-based practice has not conclusively demonstrated additional benefits from increasing the frequency or duration of exercise. Establishing dose–response relationships is crucial for enhancing the quality of evidence, as these relationships ensure the association between dose and response through objective methods, thereby facilitating the formulation of scientifically effective intervention.

Therefore, this study aimed to utilize a robust error meta-regression model (REMR) to explore the dose–response relations between exercise temporal parameters (including cycle, frequency, and duration) and cognitive function improvements in older adults with MCI. By drawing on high-quality evidence, the study seeks to provide optimal timing protocols for the treatment of MCI.

## 2 Methods

This study adheres to the Preferred Reporting Items for Systematic Reviews and Meta-Analyses of the Effects of TCEs on the Cognitive Function of Older Adults with MCI (PRISMA-A) guidelines (Supplementary material) (26). The study protocol was registered with PROSPERO ^1^ on May 29, 2024, under the registration number CRD42024510378.

### 2.1 Literature search strategy

Two researchers independently conducted a systematic search across multiple databases, including PubMed, Embase, The Cochrane Library, Web of Science, China Biology Medicine, China National Knowledge Infrastructure (CNKI), Wanfang Database, and the VIP Chinese Science and Technology Periodicals Database. From the establishment of the database to September 2024. References from review articles and included publications were manually screened to supplement the relevant literature. The search strategy combined subject terms and free-text keywords in both Chinese and English, using the following terms: (“MCI” OR “Mild Cognitive Impairment” OR “Mild Cognitive Impairment Disease” OR “Cognitive Impairment” OR “Cognitive Function”) AND (“Chinese Traditional Exercises” OR “Exercises” OR “Health Qigong” OR “Baduanjin” OR “Tai Chi” OR “Tai Chi Chuan” OR “Wuqinxi” OR “Yijinjing” OR “Liuzijue”). No restrictions were applied regarding region, language, or publication type.

### 2.2 Inclusion and exclusion criteria

#### 2.2.1 Inclusion criteria

This study follows the PICOS framework and only RCTs were included based on the following criteria:

1. Subjects were required to meet the diagnostic criteria for MCI, or refer to the diagnostic criteria established by Petersen et al. (27, 28);
2. Participants were aged ≥ 60 years;
3. The intervention group underwent a planned and organized TCEs therapy;
4. The control group received health education, engaged in other forms of exercise different from the intervention group, or maintained their original lifestyle;
5. The outcome measure was the overall cognitive function score, including the Montreal Cognitive Assessment (MoCA) and Mini-Mental State Examination (MMSE) scores (29, 30).

#### 2.2.2 Exclusion criteria

Studies with the following characteristics were excluded:

1. Studies with unclear age descriptions for the subjects;
2. Studies where the subjects’ lifestyle included regular exercise;
3. Studies that excluded MCI caused by stroke, vascular diseases, or other diseases;
4. Studies with incomplete outcome data that could not be extracted;
5. Duplicate publications, clinical protocols, case reports, review articles, and non-randomized controlled trials;
6. Studies not published in Chinese or English.

### 2.3 Data extraction

**Figure 1:**
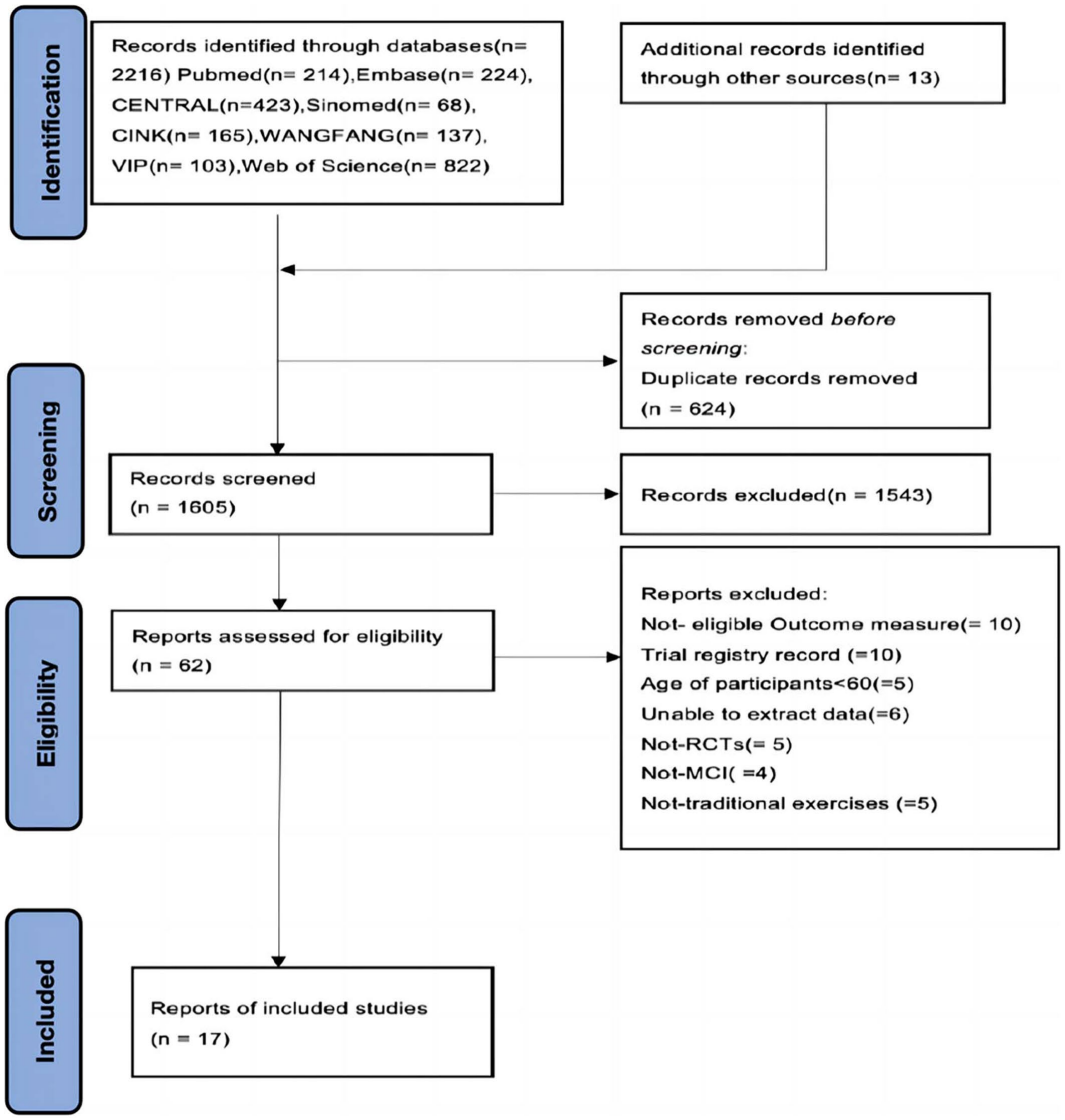
Flow diagram of the systematic review.

Duplicate literature was eliminated using EndNote 20 software. The titles and abstracts of the references were initially screened by two researchers (YL and LL), followed by a full-text review based on the inclusion and exclusion criteria. In cases of uncertainty, a third party (QL) was consulted to arbitrate. The PRISMA flowchart for literature screening is presented in Figure 1. Two researchers (QW and LL) independently extracted relevant information from the included studies. They reviewed and resolved any inconsistencies through discussion and negotiation, completing data merging and conversion (31). If any relevant information was unavailable, the corresponding authors were contacted for clarification. Data extraction was conducted using a predefined Excel sheet with the following categories: A. General Information: Literature ID, author details, year of publication, country, and participant characteristics (e.g., age, gender, sample size, and TCEs duration); B. Risk of Bias indicators: Randomization methods, blinding, and allocation concealment; C. TCEs Interventions: Details on interventions such as Baduanjin, Tai Chi, Wuqinxi, and Liuzijue, including exercise cycle, frequency, and duration; D. Outcomes: Mean and standard deviation of each post-test outcome.

### 2.4 Unification of data and dealing with missing data

Before conducting the statistical analysis, all extracted data were verified by one researcher (LL) to ensure accuracy. Corresponding authors were contacted to obtain any missing data. If the missing data could not be accurately retrieved, the study was excluded from the analysis.

### 2.5 Statistical analysis

We used R4.3.3 software, “robvis” package to assess bias risk and draw plots, and the “PEDro scale” to assess the quality of literature. The “Meta” package was used for meta-analysis, using the random-effects model (DerSimonian-Laird method), and I^2^ to measure heterogeneity quantitatively. Sensitivity analysis was performed when significant heterogeneity (I^2^ ≥ 50%) was present. The “forestplot” package was used to generate forest plots. We used Stata 15.0 software (Stata Corp., College Station, TX, United States) to analyze the dose–response relationship between TCEs exercise time parameters (cycle, frequency, and duration) and MoCA and MMSE improvements using robust error-meta regression (REMR). The effect size was estimated by the Standard Mean Difference (SMD) and 95% Confidence Interval (CI).

## 3 Results

### 3.1 Results of the literature search

A total of 2,216 records were obtained from eight databases (English and Chinese) and other meta literature, with 624 records remaining after deduplication. 1,543 literature entries were excluded after screening by titles and abstracts. After screening the full text of the remaining 62 studies, Not-eligible Outcome measure (=10), Frial registry record (=10), Age of participants<60 (=5), Unable to extract data (=6), Not-RCTs (= 5), Not-MCI (=4), Not-traditional exercises (=5). Finally, 17 eligible RCTs were included in this study, involving a total of 2,227older adults with MCI.

The articles were published between 2011 and 2023, with all 17 studies conducted in China. Seven articles were in English, and 10 were in Chinese. The study sample sizes ranged from 40 to 389, with an average of 118, and the mean age of participants was over 60 years. Table 1 summarizes the basic characteristics of the 17 studies included in the meta-analysis. None of the participants had prior experience with Traditional Chinese Exercises (TCEs) or regular exercise habits. In terms of exercise frequency, two studies did not report the number of sessions per week (22, 33), and one study did not report the duration exercise (34). The remaining studies provided complete details. Regarding outcomes, Thirteen studies included MoCA and MMSE assessments: 10 studies used MoCA outcome (19, 35–43), three studies used MMSE outcome (22, 44–46), and four studies used both MoCA and MMSE outcomes (33, 34, 47, 48).

**Table 1:** The details of the research general characteristics (*n* = 17).

Author, year (country)	Study design	Total sample size (control/experiment)	Age range (year)	Intervention		Frequency andduration	Outcomes
				Control experiment			
Chen (2017) (China) (22)	RCT Paralle	60 (30/30)	64~72	No intervention	Liuzijue	90 min/t, 3 mon	②
Cai (2018) (China) (47)	RCT Paralle	58 (30/28)	≥60	No intervention	Baduanjin Yijinjing Wuqinxi Liuzijue	90 min/t, 5 t/w, 6 mon	①, ②
Lin (2016) (China) (33)	RCT Paralle	98 (49/49)	60~73	Health education	Baduanjin	60 min/t, 6 mon	①, ②
Lin (2017) (China) (34)	RCT Paralle	94 (47/47)	61~79	Health education	Baduanjin	1 t/d, 6 t/w, 6 mon	①, ②
Liu (2018) (China) (35)	RCT Paralle	57 (29/28)	≥60	No intervention	Baduanjin	60 min/t, 6 t/w, 6 mon	①
Sun (2021) (China) (36)	RCT Paralle	57 (28/29)	65~85	Health education	Baduanjin	50 min/t, 3 t/w, 24 w	①
Xia (2020) (China) (37)	RCT Paralle	102 (51/51)	≥60	Usual physical activity	Baduanjin	60 min/t, 3 t/w, 24 w	①
Tao (2019) (China) (38)	RCT Paralle	40 (20/20)	≥60	Health education	Baduanjin	60 min/t, 3 t/w, 24 w	①
Li (2017) (China) (39)	RCT Paralle	90 (45/45)	≥60	Health education	Baduanjin	60 min/t, 3 t/w, 24 w	①
Zheng (2021) (China) (40)	RCT Paralle	40 (20/20)	≥60	Usual physical activity	Baduanjin	60 min/t, 3 t/w, 24 w	①
Zheng (2013) (China) (48)	RCT Paralle	88 (43/45)	60~77	No intervention	Liuzijue	30 min/t, 2 t/d, w ≥ 5 t, 6 m	①, ②
Lam (2011) (China) (44)	RCT Paralle	389 (218/171)	≥65	Stretching and toning exercise	Tai Chi	min/t ≥ 30, t/w ≥ 3,1 y	②
Lam (2014) (China) (45)	RCT Paralle	265 (169/96)	≥65	Stretching and relaxation exercises	Tai Chi	min/t ≥ 30, t/w ≥ 3,1 y	②
Long (2020) (China) (41)	RCT Paralle	102 (51/51)	65~85	Usual physical activity	Tai Chi	60 min/t, 4 t/w, 4 mon	①
Sui (2018) (China) (46)	RCT Paralle	160 (80/80)	≥60	Usual physical activity	Tai Chi	60 min/t, 2 t/w, 16 w	②
Li (2022) (China) (42)	RCT Paralle	46 (22/24)	≥60	Stretching	Tai Chi	60 min/t, 2 t/w, 16 w	①
Liu (2021) (China) (43)	RCT Paralle	40 (20/20)	≥60	Health education	Baduanjin	60 min/t, 3 t/w, 24 w	①

y, year; mon, month; d, day; t, time; w, week; ①, Montreal Cognitive Assessment (MoCA); ②, Mini-Mental State Examination (MMSE).

### 3.2 Risk of bias assessment

**Figure 2:**
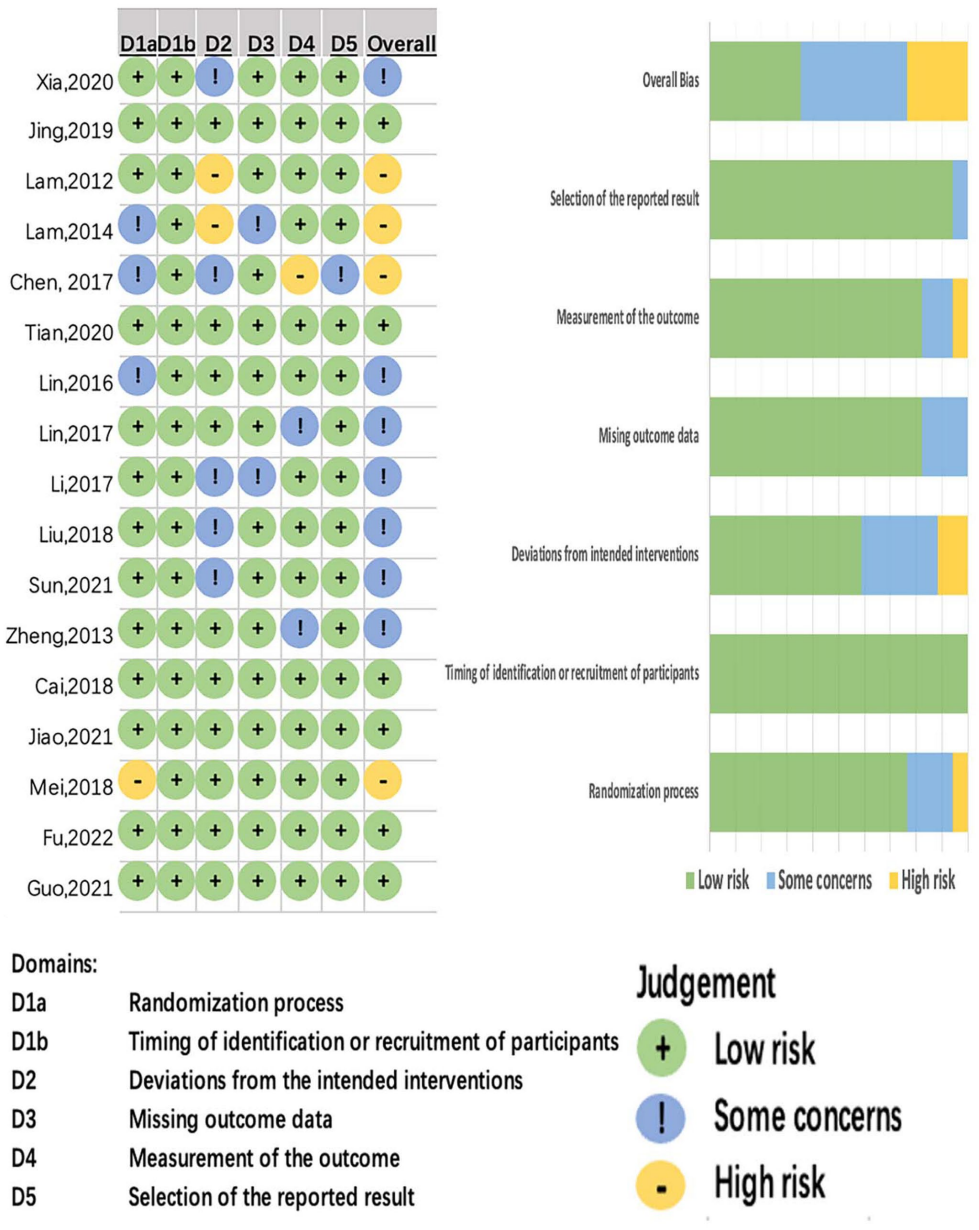
The risk of bias of included studies.

Figure 2 presents the results of the risk of bias assessment for RCTs indicating that their quality was relatively reliable. The high risk of bias was primarily due to deviations from intended interventions and the measurement of the outcome. Some concerns regarding bias were mainly due to deficiencies in the randomization process, missing outcome data, and selection of the reported result. High risk of bias was noted in four studies (22, 44–46), while seven studies demonstrated a low risk of bias (37, 38, 40–43, 47). The remaining studies all had some concerns regarding bias (19, 33–36, 39, 48).

### 3.3 Quality assessment

Table 2 describes the completeness of the details of CTEs interventions reported in accordance with the Physiotherapy Evidence Database (PEDro) guidelines. No RCTs fulfilled all reporting items. Five items were consistently reported, earning a score of 5 points: random allocation, baseline similarity, intention-to-treat analysis, between-group comparisons, and point and variability measures. Due to the nature of exercise therapy, blinding of subjects and therapists was not possible, resulting in scores of 0 points for these two items. Additionally, five studies adopted allocation concealment in their experimental design, and five studies implemented single-blind protocols for assessor blinding. A study scoring 9 or 10 points was considered of very good quality, scores of 6–8 points reflected good quality, scores of 4–5 points indicated moderate quality, and scores of 0–3 points denoted poor quality (49). The results showed that all 17 studies scored between 7 and 9 points (Table 1), indicating the included studies were of good quality.

**Table 2:** Quality assessment based on PEDro of included studies (*n* = 17).

Study	EC	RA	CA	SIB	SUB	TB	AB	>85%R	IT	BGC	PVM	TS
Chen (2017) (22)	1	1	0	1	0	0	0	1	1	1	1	7
Cai (2018) (47)	1	1	0	1	0	0	0	1	1	1	1	7
Lin (2016) (33)	1	1	0	1	0	0	0	1	1	1	1	7
Lin (2017) (34)	1	1	0	1	0	0	0	1	1	1	1	7
Liu (2018) (35)	1	1	1	1	0	0	0	1	1	1	1	8
Sun (2021) (36)	1	1	0	1	0	0	0	1	1	1	1	7
Xia (2020) (37)	1	1	1	1	0	0	1	1	1	1	1	9
Tao (2019) (38)	1	1	0	1	0	0	0	1	1	1	1	7
Li (2017) (39)	1	1	1	1	0	0	1	1	1	1	1	9
Zheng (2021) (40)	1	1	1	1	0	0	1	1	1	1	1	9
Zheng (2013) (48)	1	1	0	1	0	0	0	1	1	1	1	7
Lam (2011) (44)	1	1	0	1	0	0	1	0	1	1	1	7
Lam (2014) (45)	1	1	0	1	0	0	1	0	1	1	1	7
Long (2020) (41)	1	1	0	1	0	0	0	1	1	1	1	7
Sui (2018) (46)	1	1	0	1	0	0	0	1	1	1	1	7
Li (2022) (42)	1	1	1	1	0	0	0	1	1	1	1	8
Liu (2021) (43)	1	1	0	1	0	0	0	1	1	1	1	7

EC, Eligibility Criteria; RA, Random Allocation; CA, Concealed Allocation; SIB, Similarity Baseline; SUB, Subject Blinding; TB, Therapist Blinding; AB, Assessor Blinding; >85% R, >85% Retention; IT, Intention-to-Treat; BGC, Between-Group Comparisons; PVM, Point and Variability Measures; TS, Total Score.

### 3.4 Intervention characteristics

Table 1 provides detailed information on TCE interventions for older adults with MCI. All included studies were RCTs with sample sizes ranging from 40 to 389, with the smallest sample size being 40 and the largest 389. In the control group, four studies had no intervention (22, 35, 47, 48), four studies focused on daily exercise (37, 40, 41, 46), six studies involved health education (33, 34, 36, 38, 39, 43), and three studies included stretching exercises (42, 44, 45). In the experimental group, nine studies examined Baduanjin (33–40, 43), five studies explored Tai Chi (41, 42, 44–46), two studies investigated Liuzijue (22, 40), and one study examined a combination of TCEs (Baduanjin, Tai Chi, Wuqinxi, Liuzijue) (47).

### 3.5 Meta-analysis

#### 3.5.1 Meta-analysis of outcomes

##### 3.5.1.1 Influence on the MOCA outcome

**Figure 3:**
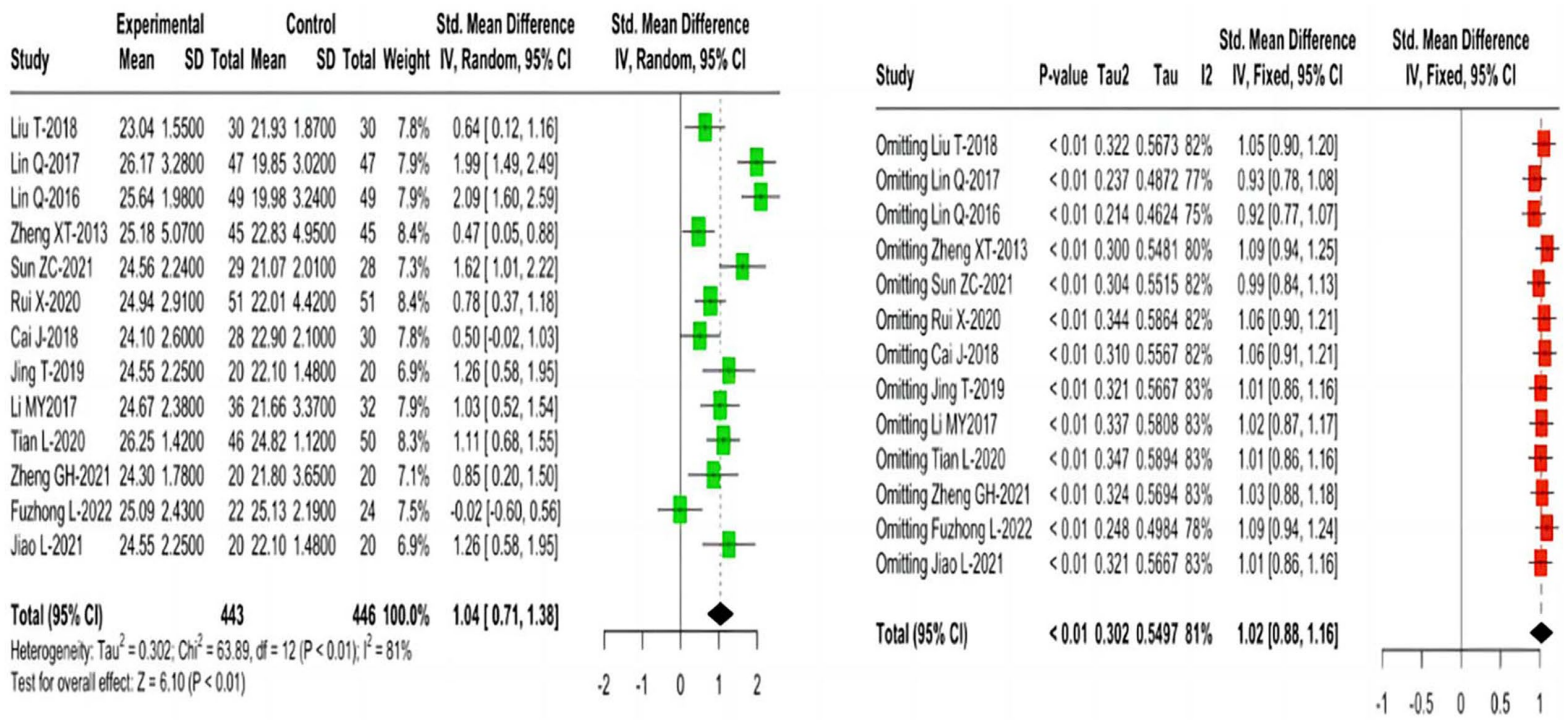
Forest plots for the effects of MoCA outcome and sensitivity analysis.

Thirteen studies reporting MoCA outcomes indicated that, compared with the control group, TCEs significantly improved cognitive function in older adults with MCI, using a random effects model (SMD = 1.04; 95% CI = 0.71 1.38; I^2^ = 81%). Sensitivity analysis demonstrated the good robustness of the result (Figure 3).

##### 3.5.1.2 Impact on the MMSE outcome

**Figure 4:**
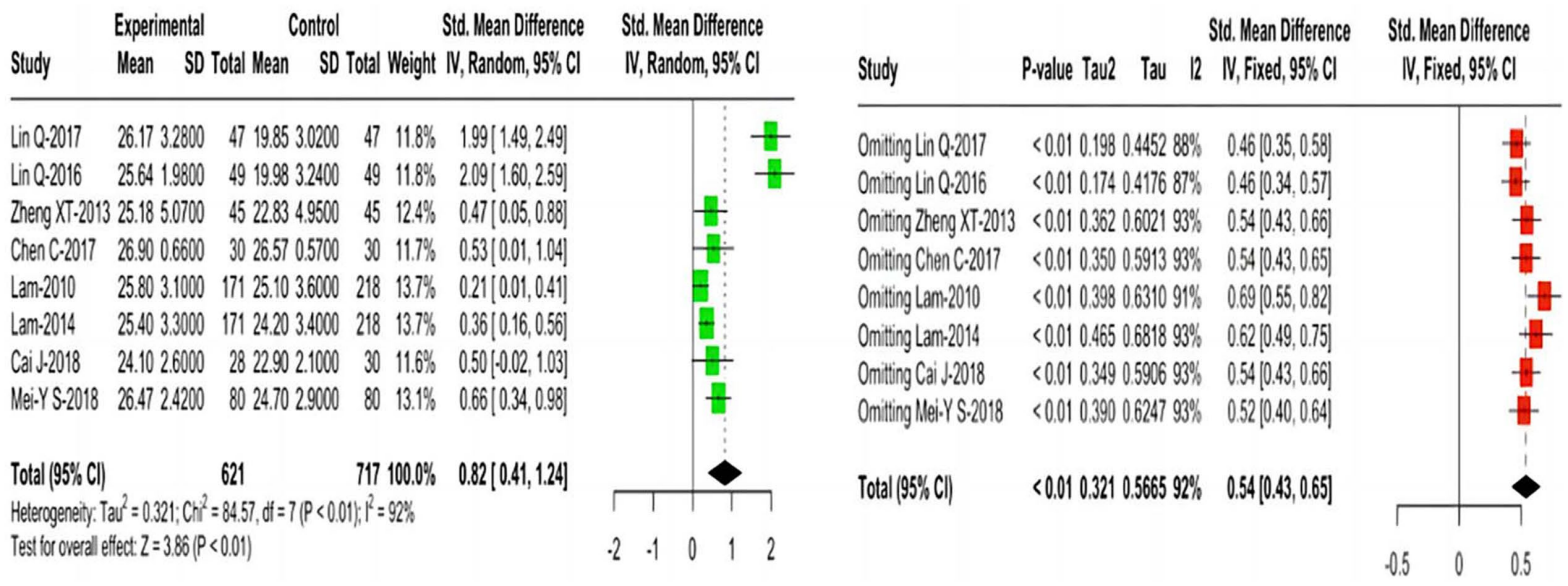
Forest plots for the effects of MMSE outcome and sensitivity analysis.

Eight studies reporting MMSE outcomes indicated that, compared with the control group, TCEs significantly improved cognitive function in older adults with MCI, using a random-effects model (SMD = 0.82; 95% CI = 0.41 1.24; I^2^ = 92%). Further sensitivity analysis demonstrated the robustness (Figure 4).

#### 3.5.2 Subgroup meta-analysis

Subgroup analyses used studies with MoCA and MMSE outcomes were conducted to investigate potential associations between efficacy and covariates (cycle, frequency, duration, and control type) (Table 3). TCEs demonstrated an overall enhancement in cognitive function. The MoCA results showed that the SMD for the experimental group was 1.28 (95% CI: 0.91–1.65) for Baduanjin, 0.57 (95% CI: −0.54–1.67) for Tai Chi, and 0.47 (95% CI: 0.05–0.88) for Liuzijue. The MMSE results revealed that the SMD for the experimental group was 2.04 (95% CI: 1.69–2.39) for Baduanjin, 0.38 (95% CI: 0.14–0.62) for Tai Chi, and 0.49 (95% CI: 0.17–0.82) for Liuzijue. For MoCA outcomes, the SMD for duration was 1.62 (95% CI: 1.01–2.22) for a practice duration of 50 min and 0.50 (95% CI: −0.02–1.03) for 90 min. For MMSE outcomes, the SMD for duration was 0.28 (95% CI: 0.13–0.43) for 30 min and 1.02 (95% CI: 0.20–1.85) for 60 min. Furthermore, the MoCA results demonstrated that the SMD for frequency ranged from −0.02 (95% CI: −0.60– 0.56) to 2.09 (95% CI: 1.60–2.59). Similarly, the MMSE results showed an SMD for frequency ranging from 0.28 (95% CI: 0.13–0.43) to 2.09 (95% CI: 1.60–2.59). The MoCA results indicated no significant statistical the experimental group (Tai Chi) (*p* = 0.32). However, variations in cycle, frequency, duration, experimental group, and control type were significantly associated with improvements in both MoCA and MMSE outcomes (*p* < 0.01).

**Table 3:** Subgroup analysis results regarding the effects of Traditional Chinese Exercises on cognition function.

Variable	No. of studies	SMD (95%CI)	*P*-value	I^2^ (%)	*p* for heterogeneity	*p* for subgroup differences
MoCA						
Overall	13	1.04 (0.71,1.38)	<0.01	81.0	<0.01	
Duration	12					0.02
50	1	1.62 (1.01,2.22)	-	-	-	
60	10	0.94 (0.71,1.48)	<0.01	78.0	<0.01	
90	1	0.50 (−0.02,1.03)	-	-	-	
Frequency	13					<0.01
2	1	−0.02 (−0.60,0.56)	-	-	-	
3	6	1.08 (0.82,1.34)	<0.01	20.0	0.29	
4	1	1.11 (0.68,1.55)	-	-	-	
5	2	0.48 (0.15,0.81)	<0.01	0.0	0.91	
6	2	1.32 (−0.01,2.64)	0.05	93.0	<0.01	
7	1	2.09 (1.60,2.59)	-	-	-	
Cycle	13					<0.01
16	1	−0.02 (−0.06,0.56)	-	-	-	
18	1	1.11 (0.68,1.55)	-	-	-	
24	7	1.00 (0.73,1.28)	<0.01	39.0	0.13	
26	4	1.29 (0.43,2.15)	<0.01	92.0	<0.01	
Experimental group	12					0.01
Baduanjin	9	1.28 (0.91,1.65)	<0.01	76.0	<0.01	
Taichi	2	0.57 (−0.54,1.67)	0.32	89	<0.01	
Liuzijue	1	0.47 (0.05,0.88)	-	-	-	
Control group	13					<0.01
Usual physical activity	6	0.76 (0.52,1.01)	<0.01	27	0.24	
Health education	6	1.50 (0.58,1.93)	<0.01	78.0	<0.01	
Stretching	1	−0.02 (−0.60,0.56)	-	-	-	
MMSE						
Overall	8	0.83 (0.32,1.34)	<0.01	92	<0.01	
Duration	7					0.13
30	2	0.28 (0.13,0.43)	<0.01	7	0.30	
60	3	1.02 (0.20,1.85)	0.02	92	<0.01	
90	2	0.52 (0.15,0.88)	<0.01	0	0.95	
Frequency	7					0.13
2	1	0.66 (0.34,0.98)				
3	2	0.28 (0.13,0.43)	<0.01	7	0.30	
5	2	0.48 (0.15,0.81)	<0.01	0	0.91	
6	1	1.99 (1.49,2.49)				
7	1	2.09 (1.60,2.59)				
Cycle	8					<0.01
13	1	0.53 (0.01,1.04)				
16	1	0.66 (0.34,0.98)				
24	1	0.50 (−0.02,1.03)				
26	3	1.51 (0.42,2.59)	<0.01	94.0	<0.01	
52	2	0.28 (0.13,0.43)	<0.01	7	0.30	
Experimental group	7					<0.01
Baduanjin	2	2.04 (1.69,2.39)	<0.01	0	0.77	
Taichi	3	0.38 (0.14,0.62)	<0.01	64	0.06	
Liuzijue	2	0.49 (0.17,0.82)	<0.01	0	0.85	
Control group	8					<0.01
Usual physical activity	4	0.56 (0.36,0.77)	<0.01	0	0.89	
Health education	2	2.04 (1.69,2.39)	<0.01	0	0.77	
Stretching	2	0.28 (0.13,0.43)	<0.01	7	0.30	

#### 3.5.3 Dose–response meta-analysis

REMR methods were used to determine the non-linear, quantity-effectiveness relation between exercise temporal parameters (cycle, frequency, and duration) and the efficacy of overall cognitive function in MCI (improvements in MoCA and MMSE).

##### 3.5.3.1 Exercise cycle

**Figure 5:**
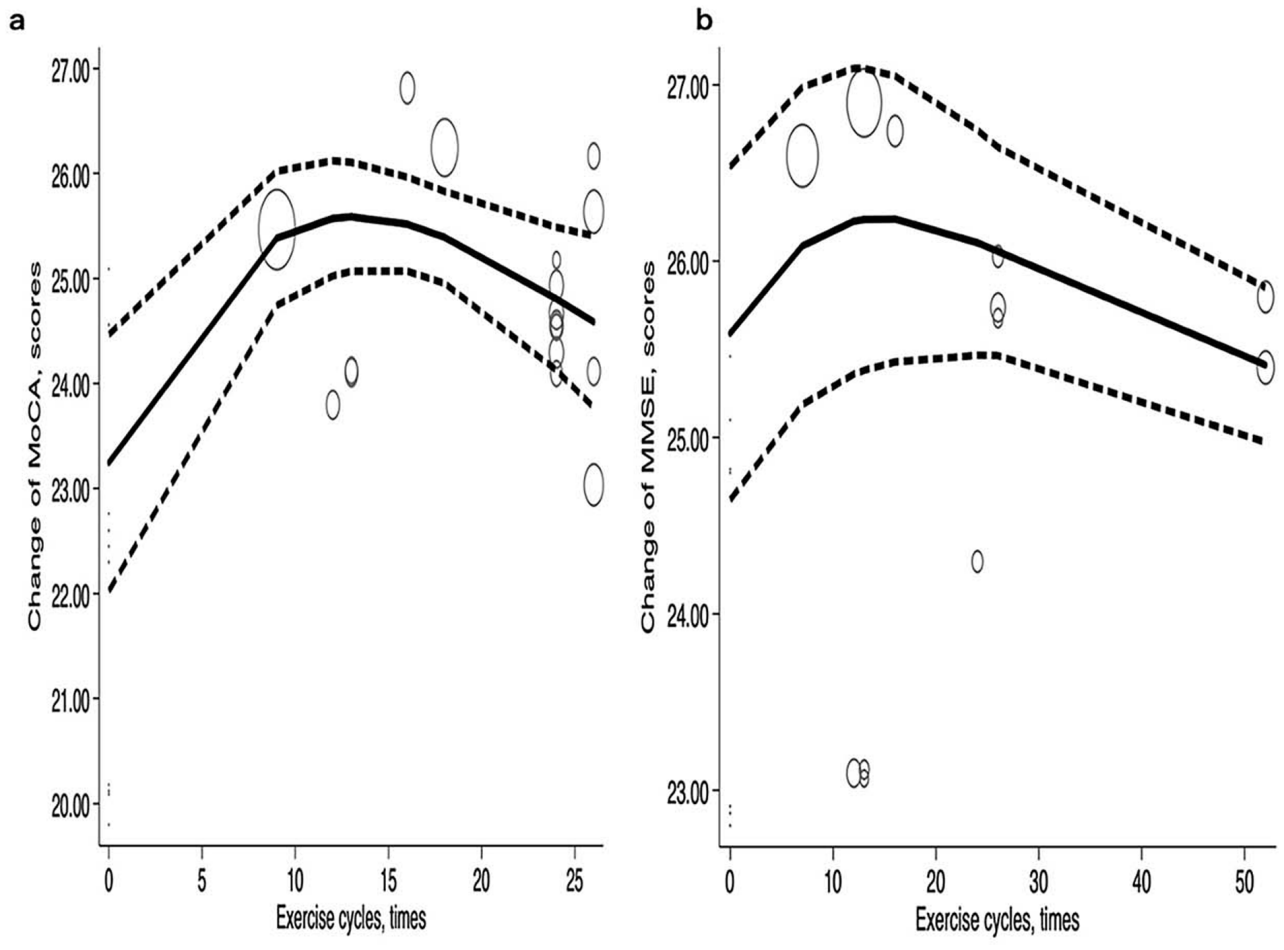
Dose–response relations between exercise cycles and changes of **(a)** MoCA and **(b)** MMSE.

The exercise cycle refers to the process of facilitating physiological adaptation in the human body through systematic and periodic adjustments of load. In this study, a one-week timeframe was established as one exercise cycle. Within a certain range, the exercise cycle was positively correlated with improvements in MoCA and MMSE, but the effect began to decline after reaching a peak, showing a ‘*Λ*’ shape. Reaching the peak may result from the cumulative enhancement of the exercise cycle and overall cognitive ability, which promotes neuroplasticity in the brain and thereby assists patients in gradually recovering their cognitive functions. After nine exercise cycles, MoCA increased to 25.38 (95% CI: 24.74–26.02), and after 12 cycles, it peaked at 25.57 (95% CI: 25.02–26.12) before beginning to decline. MoCA improvements remained stable at 25.51 (95% CI: 25.07–25.96) after 16 cycles but decreased to 24.81 (95% CI: 24.13–25.49) after 24 cycles and further to 24.59 (95% CI: 23.77–25.41). For MMSE, the score increased to 26.23 (95% CI: 25.36–27.09) after 12 cycles, peaked at 26.24 (95% CI: 25.38–27.09) after 12 cycles, and stabilized at 26.24 (95% CI: 25.43–27.05) after 16 cycles. After 24 exercise cycles, the improvement slightly decreased to 26.10 (95% CI: 25.47–26.74) (Figure 5).

##### 3.5.3.2 Exercise frequency

**Figure 6:**
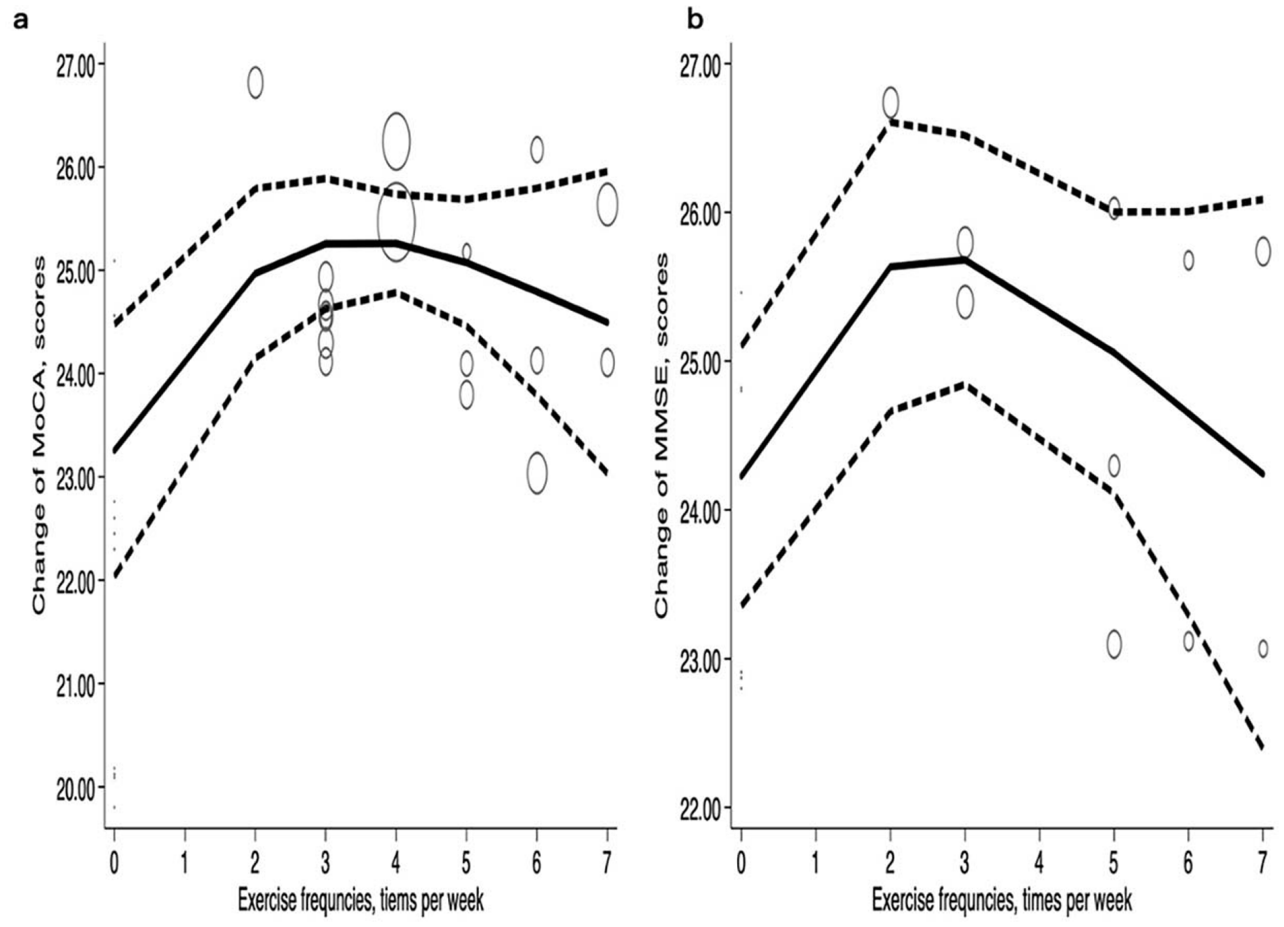
Dose–response relations between exercise frequencies and changes of **(a)** MoCA and **(b)** MMSE.

Figure 6 illustrates that with increased exercise frequency, improvements in MoCA and MMSE follow a ‘*Λ*’ pattern. Exercise frequency three times per week in a scientifically structured and appropriately tailored manner may represent a reasonable frequency for facilitating the recovery and enhancement of cognitive function in patients. The curves are similar, and the inflection points and peak values are consistent. The most significant improvements in MoCA and MMSE were observed at an exercise frequency of three times per week. Subsequently, MoCA stabilizes in a steady state, while MMSE exhibits a relatively rapid decline during the later phase. At an exercise frequency of two times per week, MoCA improved to 24.97 (95% CI: 24.15–25.79) and MMSE increased to 25.78 (95% CI: 24.66–26.61). With an exercise frequency of 3 times per week, MoCA peaked at 25.64 (95% CI: 24.62–25.89) and MMSE increased to 25.68 (95% CI: 24.84–26.52). When exercise frequency was maintained at four times per week, MoCA remained elevated at 25.26 (95% CI: 24.78–25.73), but it decreased to 24.5 (95% CI: 23.03–25.96) when exercise frequency increased to seven times per week. Additionally, the MMSE score began to decline after peaking at three times per week. An exercise frequency of seven times per week resulted in a significant decrease in the MMSE score, yielding a value of 24.25 (95% CI: 22.40–26.09).

##### 3.5.3.3 Exercise duration

**Figure 7:**
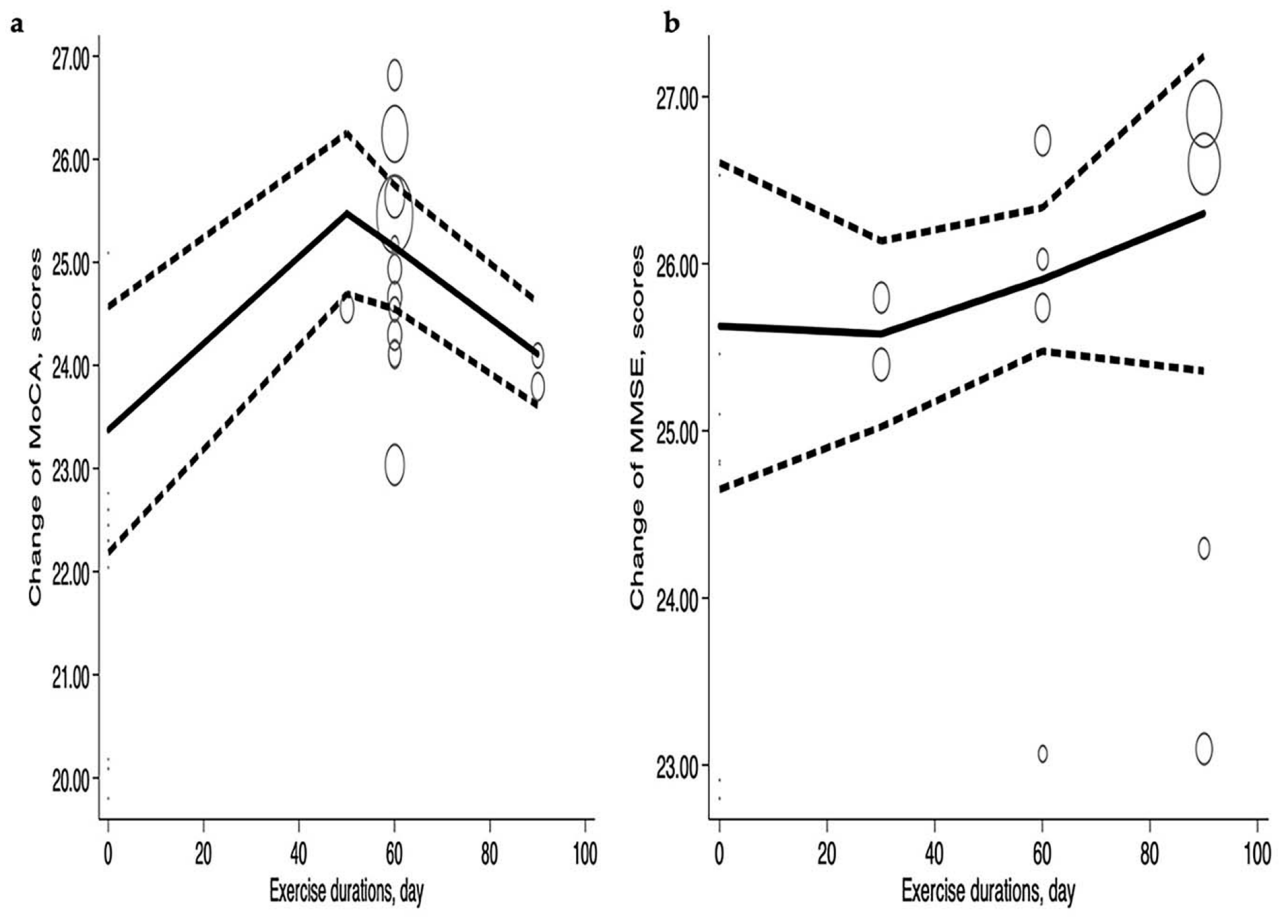
Dose–response relations between exercise durations and changes of **(a)** MoCA and **(b)** MMSE.

Figure 7 illustrates the improvement in MoCA and MMSE scores as exercise duration increases. MoCA reached its peak value of 25.47 (95% CI: 24.69–26.25) at an exercise duration of 50 min, exhibiting a ‘*Λ*’ pattern. At 30 min, MMSE showed a slight decrease before improving over time. MoCA declined slightly to 25.15 (95% CI: 24.55–25.75) at 60 min and further decreased to 24.11 (95% CI: 23.61–24.61) at 90 min. In contrast, MMSE scores increased with longer exercise durations. There was a slight improvement at 30 min, reaching 25.58 (95% CI: 25.02–26.14), and the most significant improvement occurred at 90 min, with a value of 26.3 (95% CI: 25.36–27.24). The MoCA and MMSE scores reaching the peak may result from exercise duration may ensure an adequate level of physical activity, thereby promoting the restoration of cognitive functions.

## 4 Discussion

This study exclusively conducted a dose–response meta-analysis for overall cognitive function (MoCA and MMSE), with a comprehensive analysis of the novelty associated with three temporal parameters (cycle, frequency, and duration). This approach contrasts sharply with previous studies that predominantly focused on a single parameter. Furthermore, prior meta-analyses encompassed overall cognitive function as well as specific domains such as long-term memory, short-term memory, language abilities, and conversion. On the one hand, this study failed to include indicators of exercise intensity, which may limit its comprehensiveness. On the other hand, the accuracy of the REMR effect may be influenced by the use of SMD. Furthermore, only 1–2 indicators were typically selected in exercise dose–response meta-analysis studies. Generally, at least 10 samples are required for meta-regression analysis (50). MoCA and MMSE were chosen for this study due to their robust data support in assessing older adults with MCI and their widespread application in research.

TCEs significantly enhance cognitive function in older adults, particularly in terms of overall cognition, and exhibit a significant correlation with improvements in MCI. The included studies have indicated an uneven distribution of exercise temporal parameters. The exercise cycle predominantly focused on 16 weeks and 24 weeks, with the shortest cycle being 12 weeks and the longest extending to 1 year. Exercise frequency were primarily centered around 3 times per week, ranging from a minimum of 2 to a maximum of 7 times per week. Exercise duration typically emphasized 60 min per session, with durations spanning from a minimum of 30 min to a maximum of 90 min. Furthermore, TCEs demonstrate substantial effects in older adults with MCI. The data support a strong correlation between these factors, which is largely influenced by the exercise temporal parameters. A previous meta-analysis indicated that Baduanjin exercises yield optimal results when performed for 3 months, at a frequency of 3 times per week, with each session lasting 60 min (25). However, variability in exercise temporal parameters leads to discrepancies in efficacy, complicating the effective evaluation of optimal outcomes.

The study demonstrated a nonlinear dose–response relationship between exercise temporal parameters and cognitive function outcomes in older adults with MCI. The most pronounced improvement in cognitive function, as assessed by MoCA and MMSE, was observed after 12 weeks of exercise. Furthermore, both MoCA and MMSE exhibited a positive correlation with exercise frequency, with significant enhancement observed at a frequency of three sessions per week. Although significant heterogeneity was present in the study, sensitivity analysis demonstrated strong robustness. Subgroup analyses explored the potential impact of cycle, frequency, duration, experimental type, and control type. The results indicated significant associations between these factors and the efficacy of TCEs. Previous studies have also demonstrated that TCEs significantly improve MCI. TCEs have beneficial effects on overall cognition and memory, with Baduanjin showing more pronounced effects on overall cognition compared to other TCEs (51). TCEs possess the potential to enhance both global cognitive function and various domains of cognitive function (52, 53). In the network meta-analysis, the MMSE results ranked Baduanjin exercise (78%) as the best intervention, with Tai Chi (36%) ranking second. The MoCA results ranked Baduanjin exercise (62%) as the best intervention (54). Certain scholars contend that Tai Chi is effective in enhancing cognitive function among older adults and mitigating the onset of cognitive impairment (55). As research has progressed, an increasing number of scholars have focused on the influence of temporal parameters on cognitive function. A prior meta-analysis revealed that Tai Chi exercise interventions should be last for 12 weeks, with three times per week, each lasting between 30 and 60 min (23). Others believe that Tai Chi and Qigong may improve cognitive function and increase αβ1-42 protein levels while reducing Tau protein levels in older adults with mild to moderate cognitive impairment. They recommend exercises lasting 30–90 min per session, with a frequency of 3–6 times per week, over a total cycle of 8–36 weeks (23, 24). Additionally, some scholars argue that greater benefits may be achieved through more frequent participation in group classes and home practice, ideally at least 5 times per week (56). TCEs have been shown to enhance cognitive function in older adults with MCI, with recommendations suggesting an exercise cycle of at least 3 months, a frequency of 3 times per week, and a duration of 40 min per session (25). However, there remains a lack of standardized therapeutic guidelines. Our study identified nonlinear relationships among exercise cycle, frequency, and duration, as well as their effects on outcomes. This phenomenon may be attributed to the use of different analytical models. In clinical practice, the efficacy of exercise is closely related to the accumulation of “Exercise Temporal Parameters,” and the dose–response relationship does not follow a simple linear pattern. Therefore, nonlinear models may provide a more suitable framework for understanding this relationship.

Although a previous study found a significant correlation between exercise frequency and MCI outcomes (25), our study is the first to investigate the dose–response relationships between exercise temporal parameters and MCI outcomes. Moreover, it is unique in its comprehensive evaluation of the “Exercise Temporal Parameters,” specifically cycle, frequency, and duration. Based on the previous studies, it has been observed that cognitive function outcomes fluctuate among older adults with MCI (23–25, 56). Consequently, this study employs both MoCA and MMSE to objectively evaluate and validate the robustness of the dose–response relationship. Previous research has indicated that the optimal stimulation frequency for both MoCA and MMSE is moderate, specifically three times per week. This may be due to the observation that while MoCA scores gradually decline, the decline is not entirely irreversible. Adverse reactions associated with TCEs were documented in the included studies. Nevertheless, TCEs appear to be a safe and effective intervention for enhancing cognitive function in older adults. Furthermore, TCEs have been shown to improve overall cognitive function in older adults with MCI.

## 5 Limitations and strengths

This study has several limitations. Firstly, the regional bias introduced by the fact that all studies were conducted in China may limit the generalizability of the findings to other populations. Secondly, there is significant heterogeneity among the included studies, potentially influenced by temporal factors, variations in exercise types, and individual characteristics. Therefore, caution is warranted when interpreting the aggregated results. Thirdly, the REMR analysis did not consider other aspects of cognitive function, such as long-term memory, short-term memory, language abilities, and cognitive transfer. Moreover, most of the included studies primarily reported baseline and outcome data only, which could affect the robustness of both the model and its conclusions. Finally, while our findings focus on MoCA and MMSE scores, dose–response relationships for other therapeutic outcomes may differ and warrant further investigation.

## 6 Conclusion

In summary, our study reveals a non-linear dose–response relationship between exercise duration and the enhancement of cognitive function in older adults with MCI. The most significant improvements in MoCA and MMSE scores were observed after a 12 weeks and 13 weeks intervention period. Furthermore, both MoCA and MMSE exhibit a positive correlation with exercise frequency, with an optimal regimen of 3 times per week identified as the most effective approach. In terms of exercise duration, MoCA shows the most significant improvement at 45 min, while MMSE demonstrates a gradual upward trend after 30 min. Nevertheless, additional large-scale and rigorously designed randomized controlled trials are necessary to validate these findings. Future research should also explore the underlying structural mechanisms contributing to the differential effects associated with varying exercise duration and frequency parameters.

## Data Availability

The original contributions presented in the study are included in the article/Supplementary material, further inquiries can be directed to the corresponding author.

## Footnotes

^1^ http://www.crd.york.ac.uk/PROSPERO
