# Supplementary material for: Optimal exercise temporal parameters of Traditional Chinese Exercises for cognitive function of older adults with mild cognitive impairment: a systematic review and dose–response meta-analysis of randomized controlled trials
Source: Front Med (Lausanne). 2025 May 7;12:1568835. doi: 10.3389/fmed.2025.1568835 (PMC12092424; doi:10.3389/fmed.2025.1568835)
Supplement: Supplementary file 1 [file Table_1.docx]

| **Section and Topic** | **Item #** | **Checklist item** | **Location where item is reported** |
| --- | --- | --- | --- |
| **TITLE** | | |  |
| Title | 1 | Identify the report as a systematic review and meta-analysis | 1 |
| **ABSTRACT** | | |  |
| Abstract | 2 | Provide a structured summary including, as applicable: background; objectives; data sources; study eligibility criteria, participants, and interventions; study appraisal and synthesis methods; results limitations; conclusions and implications of key findings; systematic review registration number. | 2 |
| **INTRODUCTION** | | |  |
| Rationale | 3 | Described in the background effect of traditional Chinese exercises (TCEs) on older adults with Mild Cognitive Impairment; If applicable, state the known traditional Chinese exercises to be studied and describe whether there are any differences in the frequency of exercises between the different types of exercises. | 5 |
| Objectives | 4 | Provide an explicit statement of questions being addressed with reference to participants, interventions, comparisons, outcomes, and study design (PICOS) | 5 |
| **METHODS** | | |  |
| Eligibility criteria | 5 | Inclusion criteria:This study follows the PICOS framework and only Randomized Controlled Trials (RCTs) were included; (1) Subjects were required to meet the diagnostic criteria for MCI, or refer to the diagnostic criteria of Petersen et al; (2) aged 60 years or older; (3) The intervention group was only a planned and organized TCEs therapy. (4) The control group was the health education group, other forms of exercise different from the intervention group or maintains the original lifestyle; (5) The outcome measure was the overall cognitive function score, including the Montreal Cognitive Assessment Scale (MoCA) score and the Simple Mental State Scale (MMES) score.  Exclusion criteria: Literature with the following characteristics was excluded: (1) The study with unclear age descriptions for subjects; (2) The subjects' lifestyle included regular exercise; (3) The study with a sample size of less than 20 cases in each group excluded mild cognitive impairment caused by stroke, vascular diseases, and other diseases; (4) The study with incomplete outcome data and which cannot be extracted; (5) Duplicate publications, clinical protocols, case reports, review articles, and non-randomized controlled trials. | 5 |
| Information sources | 6 | Describe all sources of information (e.g., databases with dates of coverage, contact with study authors to identify additional studies) in the search, and report the date of the last search. If applicable, report the databases or TCEs search methods for exercise therapy or traditional medicine. | 4 |
| Search strategy | 7 | This study follows the preferred reporting Items for systematic reviews and meta-analyses of the effects of CTEs on the cognitive function of older adults with MCI (PRISMA-A) statement (Supplement 1). | 4 |
| Selection process | 8 | A total of 1959 records were obtained from eight English and Chinese databases and other meta-literature, with 591 records remaining after de-duplication. 1311 literature entries were excluded after screening by titles and abstracts. | 7 |
| Data collection process | 9 | Two researchers (QPW and LL) independently extracted relevant information from the included literature. The same two researchers reviewed and negotiated any inconsistencies and completed data merging and conversion. | 5\6 |
| Data items | 10a | Duplicate literature was eliminated through Endnote 20 software. | 5 |
|  | 10b | The same two researchers reviewed and negotiated any inconsistencies and completed data merging and conversion. If related information was unknown, the corresponding authors w were contacted to resolve it. | 5 |
| Study risk of bias assessment | 11 | shows the results of the RoB assessment for randomized controlled trials were presented, and their quality was relatively reliable. Two reviewers assessed each study and whether they worked independently, and if applicable, details of automation tools used in the process. | 9 |
| Effect measures | 12 | The effect size was estimated by the Weighted Mean Difference (WMD) and 95% Confidence Interval (CI). | 6\10\13\14 |
| Synthesis methods6 | 13a | After screening the full text of the remaining 57 studies, Not- eligible Outcome measure (=10), Frial registry record (=10), The per group sample size <20 (=5), Age of participants<60(=5), Unable to extract data(=3), Not-RCTs(= 4)，Not-MCI( =5), Not-traditional exercises (=2). Finally, 13 eligible RCTs were included in this study, involving a total of 1532 Older Adults with MCI (Figure 1). | 7 |
|  | 13b | We contacted the corresponding authors to obtain missing data. If the data could not be accurately obtained, it was excluded. | 6 |
|  | 13c | Fig. 2 shows the results of the RoB assessment for randomized controlled trials were presented, and their quality was relatively reliable.  Tab2 describes the completeness of the details of CTEs interventions reported in accordance with the PEDro guidelines. | 10 |
|  | 13d | We used R4.3.3 software (R Core Team, 2024), "robvis" package to assess bias risk and draw plots, and the "PEDro scale" to assess the quality of literature. | 6 |
|  | 13e | Describe any methods used to explore possible causes of heterogeneity among study results (e.g. subgroup analysis, meta-regression). | 14-17 |
|  | 13f | The "Meta" package was used for meta-analysis, using the random-effects model (DerSimonian-Laird method), and I^2^ to measure heterogeneity quantitatively. Sensitivity analysis was performed when significant heterogeneity (I^2^ ≥ 50%) was present. | 6 |
| Reporting bias assessment | 14 | shows the results of the RoB assessment for randomized controlled trials were presented, and their quality was relatively reliable. | 9 |
| Certainty assessment | 15 | We used R4.3.3 software (R Core Team, 2024), "robvis" package to assess bias risk and draw plots, and the "PEDro scale" to assess the quality of literature.  The "forestplot" package was used to generate forest plots. | 6 |
| **RESULTS** | | |  |
| Study selection | 16a | A total of 1959 records were obtained from eight English and Chinese databases and other meta-literature, with 591 records remaining after de-duplication. | 7\8 |
|  | 16b | Cite studies that might appear to meet the inclusion criteria, but which were excluded, and explain why they were excluded: After screening the full text of the remaining 57 studies, Not- eligible Outcome measure (=10), Frial registry record (=10), The per group sample size <20 (=5), Age of participants<60(=5), Unable to extract data(=3), Not-RCTs(= 4)，Not-MCI( =5), Not-traditional exercises (=2). | 7 |
| Study characteristics | 17 | Tab. 2 presented the basic characteristics of the 13 studies included in the meta-analysis. | 7\9 |
| Risk of bias in studies | 18 | Present assessments of risk of bias for each included study. | 10 |
| Results of individual studies | 19 | For all outcomes, present, for each study: (a) summary statistics for each group (where appropriate) and (b) an effect estimate and its precision (e.g. confidence/credible interval), ideally using structured tables or plots. | 13\14\16 |
| Results of syntheses | 20a | High risk of bias was noted in 3 studies, while 3 studies demonstrated a low risk of bias. The rest of the studies all had "some concerns" of bias. | 9\10 |
|  | 20b | 10 studies reporting MoCA outcome indicated that compared with the control group, TCEs improved cognitive function more significantly in elderly people with MCI, using a random effects model, using a random effects model (MD=2.86; 95% CI = 1.95 4.13; I2=91%).7 studies reporting MMES outcome indicated that compared with the control group, TCEs improved cognitive function more significantly in elderly people with MCI, using a random effects model (MD=1.41; 95% CI = 0.62 2.20; I2=81%). | 12\13 |
|  | 20c | Present results of all investigations of possible causes of heterogeneity among study results. | 12\13 |
|  | 20d | Present results of all sensitivity analyses conducted to assess the robustness of the synthesized results. | 12\13 |
| Reporting biases | 21 | Present assessments of risk of bias due to missing results (arising from reporting biases) for each synthesis assessed. | 10 |
| Certainty of evidence | 22 | Present assessments of certainty (or confidence) in the body of evidence for each outcome assessed. | 11 |
| **DISCUSSION** | | |  |
| Discussion | 23a | Provide a general interpretation of the results in the context of other evidence. | 17 |
|  | 23b | Discuss any limitations of the evidence included in the review. | 18 |
|  | 23c | Firstly, the studies included were only in Chinese and English. The sample size of the included studies was relatively small, which can bias the results. Most studies did not report blindness, which could have resulted in selective bias that affected the results. Secondly, The heterogeneity between some studies is large, which may affect the reliability of meta-analyses. Thirdly, Differences in the personnel trained on the interventions may affect the reliability of the results. | 18 |
|  | 23d | In general, TCEs may become an auxiliary rehabilitation method for MCI older adults, but further clinical research is needed to confirm its specific effects. The advantage of this study is that only RCT studies with a rigorous study design were included, ensuring the quality of the included data. | 18 |
| **OTHER INFORMATION** | | |  |
| Registration and protocol | 24a | Wen Qingpan, Gdansk University of Physical Education and Sport; The protocol was registered in PROSPERO with the registration number CRD42024510378. | 4 |
|  | 24b | Indicate where the review protocol can be accessed, or state that a protocol was not prepared. if available, http://www. crd.york.ac.uk/PROSPERO. | 4 |
|  | 24c | Describe and explain any amendments to information provided at registration or in the protocol. | 4 |
| Support | 25 | Describe sources of financial or non-financial support for the review, and the role of the funders or sponsors in the review. | 19 |
| Competing interests | 26 | Declare any competing interests of review authors. | 19 |
| Availability of data, code and other materials | 27 | Report which of the following are publicly available and where they can be found: template data collection forms; data extracted from included studies; data used for all analyses; analytic code; any other materials used in the review. if available,Template data collection forms; data extracted from included studiesare found publicly, on pages 9 and 11. | 9\11\ |

*From:*  Page MJ, McKenzie JE, Bossuyt PM, Boutron I, Hoffmann TC, Mulrow CD, et al. The PRISMA 2020 statement: an updated guideline for reporting systematic reviews. BMJ 2021;372:n71. doi: 10.1136/bmj.n71 For more information, visit: <http://www.prisma-statement.org/>
