# Supplementary material for: Optimal exercise temporal parameters of Traditional Chinese Exercises for cognitive function of older adults with mild cognitive impairment: a systematic review and dose–response meta-analysis of randomized controlled trials
Source: Front Med (Lausanne). 2025 May 7;12:1568835. doi: 10.3389/fmed.2025.1568835 (PMC12092424; doi:10.3389/fmed.2025.1568835)
Supplement: Supplementary file 2 [file Table_2.docx]

Supplement. Excluded References

eTab. 3. Details of excluded References

| No. | References | Excluding Reasons |
| --- | --- | --- |
| 1 | Birimoglu Okuyan C, Deveci E. The effectiveness of Tai Chi Chuan on fear of movement, prevention of falls, physical activity, and cognitive status in older adults with mild cognitive impairment: A randomized controlled trial. Perspect Psychiatr Care. 2021 Jul;57(3):1273-1281. doi: 10.1111/ppc.12684. Epub 2020 Nov 13. Erratum in: Perspect Psychiatr Care. 2022 Oct;58(4):3118. PMID: 33184928. | Not- eligible Outcome measure |
| 2 | Chang JY, Tsai PF, Beck C, Hagen JL, Huff DC, Anand KJ, Roberson PK, Rosengren KS, Beuscher L. The effect of tai chi on cognition in elders with cognitive impairment. Medsurg Nurs. 2011 Mar-Apr;20(2):63-9; quiz 70. PMID: 21560956; PMCID: PMC3320763. | Unable to extract data |
| 3 | Efficacy of a three-month Tai-Chi programme on functional performance for Chinese community-dwelling elders with mild cognitive impairment | Not- eligible Outcome measure |
| 4 | The effect of Baduanjin exercise on cognitive function of older adults with mild cognitive impairment: a randomized controlled trial | Trial registry record |
| 5 | Effects of Tai Chi on cognition and instrumental activities of daily living in community dwelling older people with mild cognitive impairment | Trial registry record |
| 6 | The mechanism of Tai Chi Chuan improving cognitive function in patients with mild cognitive impairment -multiple model MRI study | Trial registry record |
| 7 | Effect of Ba-Duan-Jin exercise on cognitive function in patients with mild cognitive impairment | Trial registry record |
| 8 | Active and passive intervention conbined with characteristic treatment regimens of traditional Chinese medicine in mild cognitive impairment: a multicentre, randomized control study | Trial registry record |
| 9 | Effect of physical exercise intervention on the older adults with mild cognitive impairment | Trial registry record |
| 10 | Effect of Tai Chi on fall prevention and cognitive function in older adults with mild cognitive impairment | Trial registry record |
| 11 | Effects of home-based computerized cognitive training and tai chi exercise on cognitive functions in older adults with mild cognitive impairment | Not- eligible Outcome measure |
| 12 | Interim follow-up of a randomized controlled trial comparing Chinese style mind body (Tai Chi) and stretching exercises on cognitive function in subjects at risk of progressive cognitive decline | Not- eligible Outcome measure |
| 13 | The effect of 12 weeks of Baduanjin exercise on cognitive function, lower limb balance and quality of life of the elderly with mild cognitive impairment: a randomized controlled trial | Age of participants<60 |
| 14 | Liu CL, Cheng FY, Wei MJ, Liao YY. Effects of Exergaming-Based Tai Chi on Cognitive Function and Dual-Task Gait Performance in Older Adults With Mild Cognitive Impairment: A Randomized Control Trial. Front Aging Neurosci. 2022 Mar 15;14:761053. doi: 10.3389/fnagi.2022.761053. PMID: 35370622; PMCID: PMC8965318. | Not-traditional exercises |
| 15 | Tai Ji Quan and Cognitive Function in Older Adults With Mild Cognitive Impairment | Trial registry record |
| 16 | Effectiveness of Tai Chi to Improve Cognitive Function in Older Adults With Mild Cognitive Impairment | Trial registry record |
| 17 | Sungkarat S, Boripuntakul S, Chattipakorn N, Watcharasaksilp K, Lord SR. Effects of Tai Chi on Cognition and Fall Risk in Older Adults with Mild Cognitive Impairment: A Randomized Controlled Trial. J Am Geriatr Soc. 2017 Apr;65(4):721-727. doi: 10.1111/jgs.14594. Epub 2016 Nov 22. PMID: 27874176. | Not- eligible Outcome measure |
| 18 | Sungkarat S, Boripuntakul S, Kumfu S, Lord SR, Chattipakorn N. Tai Chi Improves Cognition and Plasma BDNF in Older Adults With Mild Cognitive Impairment: A Randomized Controlled Trial. Neurorehabil Neural Repair. 2018 Feb;32(2):142-149. doi: 10.1177/1545968317753682. Epub 2018 Jan 20. PMID: 29353543. | Not- eligible Outcome measure |
| 19 | Effects of exergaming based Tai Chi on Cognition and dual task gait in older adults with mild cognitive Impairment: a randomized control trial | Trial registry record |
| 20 | Yu AP, Chin EC, Yu DJ, Fong DY, Cheng CP, Hu X, Wei GX, Siu PM. Tai Chi versus conventional exercise for improving cognitive function in older adults: a pilot randomized controlled trial. Sci Rep. 2022 May 25;12(1):8868. doi: 10.1038/s41598-022-12526-5. PMID: 35614144; PMCID: PMC9131984. | Age of participants<60 |
| 21 | Zheng G, Huang M, Li S, Li M, Xia R, Zhou W, Tao J, Chen L. Effect of Baduanjin exercise on cognitive function in older adults with mild cognitive impairment: study protocol for a randomised controlled trial. BMJ Open. 2016 Apr 11;6(4):e010602. doi: 10.1136/bmjopen-2015-010602. PMID: 27067894; PMCID: PMC4838712. | Unable to extract data |
| 22 | 刘涛,白石,黄悦,张荣超.运动干预对轻度认知障碍患者认知水平和脑脊液相关指标的影响[J].陕西医学杂志,2015,(10):1388-1390.DOI:10.3969/j.issn.1000-7377.2015.10.052. | Age of participants<60 |
| 23 | 包娜娜,刘超.太极拳对遗忘型轻度认知功能障碍患者认知功能影响的研究[J].医学信息,2019,32(2):115-117.DOI:10.3969/j.issn.1006-1959.2019.02.032. | Age of participants<60 |
| 24 | 孙建平,唐伟,王久武.运动干预对轻度认知障碍患者认知水平和脑脊液相关指标的影响[J].家庭医药.就医选药,2016(05):93-94. | Age of participants<60 |
| 25 | 崔永胜,杨慧馨.太极拳与快走锻炼对轻度认知功能障碍老年人认知功能的影响[J].哈尔滨体育学院学报,2019,37(05):17-22. | Not-RCTs |
| 26 | 潘静亚.认知训练联合运动疗法对老年轻度认知障碍患者认知功能的影响[J].健康必读,2020,(4):82. | Not-traditional exercises |
| 27 | 王乾贝. 太极拳运动对社区轻度认知障碍老年人认知功能的影响[D].北京协和医学院,2017. | Not-RCTs |
| 28 | 王乾贝,绳宇.太极拳运动对社区轻度认知障碍老年人认知功能的影响[J].中国康复理论与实践,2016,22(06):645-649. | Not-RCTs |
| 29 | 王欣. 健身气功八段锦对轻度认知功能障碍者脑电波的影响[D].沈阳体育学院,2020. | Unable to extract data |
| 30 | 田龙.太极拳对MCI老年人认知功能与执行功能的影响[J].承德医学院学报,2020,37(02): 173-176.DOI: 10.15921/j.cnki.cyxb.2020.02.031. | Not-RCTs |
| 31 | 郭晋瑜,赵娅蓉,吕亮亮等.一种新型认知训练方法对轻度认知障碍患者各认知域功能的影响[J].神经损伤与功能重建,2022,17(01): 17-22.DOI: 10.16780/j.cnki.sjssgncj.20210127. | Not-traditional exercises |
| 32 | 徐志立,张莹,张旭,王华.八段锦对老年人智力衰老的改善作用研究[J].亚太传统医药,2019(2). | Not-RCTs |
| 33 | Deschamps A, Onifade C, Decamps A, Bourdel-Marchasson I. Health-related quality of life in frail institutionalized elderly: effects of a cognition-action intervention and Tai Chi. J Aging Phys Act. 2009 Apr;17(2):236-48. doi: 10.1123/japa.17.2.236. PMID: 19451671. | Not-MCI |
| 34 | Birimoglu Okuyan C, Deveci E. The effectiveness of Tai Chi Chuan on fear of movement, prevention of falls, physical activity, and cognitive status in older adults with mild cognitive impairment: A randomized controlled trial. Perspect Psychiatr Care. 2021 Jul;57(3):1273-1281. doi: 10.1111/ppc.12684. Epub 2020 Nov 13. Erratum in: Perspect Psychiatr Care. 2022 Oct;58(4):3118. PMID: 33184928. | Not-MCI |
| 35 | 孙皎,王黎,张秀英,嫣红,李洪艳.太极拳运动对老年人脑功能及体力的改善作用[J].中国老年学杂志,2011,31(23):4688-4689.DOI:10.3969/j.issn.1005-9202.2011.23.091. | Not-MCI |
| 36 | Siu MY, Lee DTF. Is Tai Chi an effective intervention for enhancing health-related quality of life in older people with mild cognitive impairment? An interventional study. Int J Older People Nurs. 2021 Sep;16(5):e12400. doi: 10.1111/opn.12400. Epub 2021 Jul 13. PMID: 34254731. | Not-MCI |
| 37 | 傅经明, et al.八段锦对天津市老年人群认知功能的干预作用.中国老年学杂志 38.03(2018):632-634. | Not- eligible Outcome measure |
| 38 | Sungkarat S, Boripuntakul S, Kumfu S, Lord SR, Chattipakorn N. Tai Chi Improves Cognition and Plasma BDNF in Older Adults With Mild Cognitive Impairment: A Randomized Controlled Trial. Neurorehabil Neural Repair. 2018 Feb;32(2):142-149. doi: 10.1177/1545968317753682. Epub 2018 Jan 20. PMID: 29353543. | Not- eligible Outcome measure |
| 39 | Sungkarat S, Boripuntakul S, Chattipakorn N, Watcharasaksilp K, Lord SR. Effects of Tai Chi on Cognition and Fall Risk in Older Adults with Mild Cognitive Impairment: A Randomized Controlled Trial. J Am Geriatr Soc. 2017 Apr;65(4):721-727. doi: 10.1111/jgs.14594. Epub 2016 Nov 22. PMID: 27874176. | Not- eligible Outcome measure |
| 40 | Fogarty JN, Murphy KJ, McFarlane B, Montero-Odasso M, Wells J, Troyer AK, Trinh D, Gutmanis I, Hansen KT. Taoist Tai Chi® and Memory Intervention for Individuals with Mild Cognitive Impairment. J Aging Phys Act. 2016 Apr;24(2):169-80. doi: 10.1123/japa.2014-0062. Epub 2015 Apr 2. PMID: 25838271. | Not- eligible Outcome measure |
| 41 | A Study on the Effect of Traditional Chinese Exercise Combined With Rhythm Training onthe Intervention of Older Adults With MildCognitive Impairment | Not-traditional exercises |
| 42 | Xia R, Wan M, Lin H, Ye Y, Chen S, Zheng G. Effects of mind-body exercise Baduanjin on cognition in community-dwelling older people with mild cognitive impairment: A randomized controlled trial. Neuropsychol Rehabil. 2023 Sep;33(8):1368-1383. doi: 10.1080/09602011.2022.2099909. Epub 2022 Jul 15. PMID: 35838817. | Not-traditional exercises |
| 43 | Effect of 1 Year of Qigong Exercise on Cognitive Function Among Older Chinese Adults at Risk of Cognitive Decline: A Cluster Randomized Controlled Trial | Unable to extract data |
| 44 | Effects of a traditional Chinese mind–body exercise, Baduanjin, on the physical and cognitive functions in the community of older adults with cognitive frailty: study protocol for a randomised controlled trial | Unable to extract data |
| 45 | Sungkarat S, Boripuntakul S, Kumfu S, Lord SR, Chattipakorn N. Tai Chi Improves Cognition and Plasma BDNF in Older Adults With Mild Cognitive Impairment: A Randomized Controlled Trial. Neurorehabil Neural Repair. 2018 Feb;32(2):142-149. doi: 10.1177/1545968317753682. Epub 2018 Jan 20. PMID: 29353543. | Unable to extract data |
