# Supplementary material for: Optimal exercise temporal parameters of Traditional Chinese Exercises for cognitive function of older adults with mild cognitive impairment: a systematic review and dose–response meta-analysis of randomized controlled trials
Source: Front Med (Lausanne). 2025 May 7;12:1568835. doi: 10.3389/fmed.2025.1568835 (PMC12092424; doi:10.3389/fmed.2025.1568835)
Supplement: Supplementary file 3 [file Table_3.docx]

Supplement. Search equations

| eTab. 2.a Search strategy for PubMed database |
| --- |
| **#1 ("Cognitive Dysfunction"[Mesh])** |
| **#2** **(((((****(((((((((((((((((((((("Cognitive Dysfunction") ) OR (Cognitive Dysfunctions[Title/Abstract])) OR (Dysfunction, Cognitive[Title/Abstract])) OR (Dysfunctions, Cognitive[Title/Abstract])) OR (Cognitive Impairments[Title/Abstract])) OR (Cognitive Impairment[Title/Abstract])) OR (Impairment, Cognitive[Title/Abstract])) OR (Impairments, Cognitive[Title/Abstract])) OR (Cognitive Disorder[Title/Abstract])) OR (Cognitive Disorders[Title/Abstract])) OR (Disorder, Cognitive[Title/Abstract])) OR (Disorders, Cognitive[Title/Abstract])) OR (Mild Cognitive Impairment[Title/Abstract])) OR (Cognitive Impairment, Mild[Title/Abstract])) OR (Cognitive Impairments, Mild[Title/Abstract])) OR (Impairment, Mild Cognitive[Title/Abstract])) OR (Impairments, Mild Cognitive[Title/Abstract])) OR (Mild Cognitive Impairments[Title/Abstract])) OR (Cognitive Decline[Title/Abstract])) OR (Cognitive Declines[Title/Abstract])) OR (Decline, Cognitive[Title/Abstract])) OR (Declines, Cognitive[Title/Abstract])) OR (Mental Deterioration[Title/Abstract])) OR (Deterioration, Mental[Title/Abstract])) OR (Deteriorations, Mental[Title/Abstract])) OR (Mental Deteriorations[Title/Abstract]))** |
| #3 **("Tai Chi "[Mesh])** |
| **#4** **(((((((((("Tai Chi ")OR (Tai-ji[Title/Abstract])) OR (Chi, Tai[Title/Abstract])) OR (Tai Ji Quan[Title/Abstract])) OR (Ji Quan, Tai[Title/Abstract])) OR (Quan, Tai Ji[Title/Abstract])) OR (Taiji[Title/Abstract])) OR (Taijiquan[Title/Abstract])) OR (T'ai Chi[Title/Abstract])) OR (Tai Chi Chuan[Title/Abstract]))** |
| #5 #2 and #4 |
| **#6** **(Baduanjin[Title/Abstract])** |
| #7 #2 and #6 |
| #8 **(Wuqinxi[Title/Abstract])** |
| #9#2 and #8 |
| **#10(Liuzijue[Title/Abstract])** |
| #11 #2 and #10 |
| #12 **(Yijinjing[Title/Abstract])** |

| eTab. 2.b Search strategy for Embase database |
| --- |
| #1 'cognitive dysfunctions':ab,ti OR 'dysfunction, cognitive':ab,ti OR 'dysfunctions, cognitive':ab,ti OR 'cognitive impairments':ab,ti OR 'cognitive impairment':ab,ti OR 'impairment, cognitive':ab,ti OR 'impairments, cognitive':ab,ti OR 'cognitive disorder':ab,ti OR 'cognitive disorders':ab,ti OR 'disorder, cognitive':ab,ti OR 'disorders, cognitive':ab,ti OR 'mild cognitive impairment':ab,ti OR 'cognitive impairment, mild':ab,ti OR 'cognitive impairments, mild':ab,ti OR 'impairment, mild cognitive':ab,ti OR 'impairments, mild cognitive':ab,ti OR 'mild cognitive impairments':ab,ti OR 'cognitive decline':ab,ti OR 'cognitive declines':ab,ti OR 'decline, cognitive':ab,ti OR 'declines, cognitive':ab,ti OR 'mental deterioration':ab,ti OR 'deterioration, mental':ab,ti OR 'deteriorations, mental':ab,ti OR 'mental deteriorations':ab,ti |
| #2 'tai ji':ab,ti OR 'chi, tai':ab,ti OR 'tai ji quan':ab,ti OR 'ji quan, tai':ab,ti OR 'quan,tai ji':ab,ti OR taiji:ab,ti OR taijiquan:ab,ti OR 'tai chi':ab,ti OR 'tai chi chuan':ab,ti |
| #3 #1 AND #2 |
| #4 baduanjin:ab,ti |
| #5 #1 AND #4 |
| #6 wuqinxi:ab,ti |
| #7 #1 AND #6 |
| #8 liuzijue:ab,ti |
| #9 #1 AND #8 |
| #10 yijinjing:ab,ti |
| #11 #1 AND #10 |

| eTab. 2.c Search strategy for The Cochrane Library |
| --- |
| #1 MeSH: [Cognitive Dysfunction] |
| #2 (Cognitive Dysfunctions or Dysfunction, Cognitive or Dysfunctions, Cognitive or Cognitive Impairments or Cognitive Impairment or Impairment, Cognitive or Impairments, Cognitive or Cognitive Disorder or Cognitive Disorders or Disorder, Cognitive or Disorders, Cognitive or Mild Cognitive Impairment or Mild Cognitive Impairment or Cognitive Impairment, Mild or Cognitive Impairments, Mild or Impairment, Mild Cognitive or Impairments, Mild Cognitive or Mild Cognitive Impairments or Cognitive Decline or Cognitive Declines or Decline, Cognitive or Declines, Cognitive or Mental Deterioration or Deterioration, Mental or Deteriorations, Mental or Mental Deteriorations) |
| #3 #1 or #2 |
| #4 MeSH [Tai Ji] |
| #5 (Tai Chi or Chi, Tai or Tai Ji Quan or Ji Quan, Tai or Quan,Tai Ji or Taiji or Taijiquan or T'ai Chi or Tai Chi Chuan) |
| #6 #4 or #5 |
| #7 #3 and #6 |
| #8 (Baduanjin) |
| #9 #3 and #8 |
| #10 (Wuqinxi) |
| #11#3 and #10 |
| #12 (Yijinjing) |
| #13 #3 and #12 |
| #14 (Liuzijue) |
| #15 #3 and #14 |

| eTab. 2.d Search strategy for Web of Science |
| --- |
| **#1 ( TS=(Cognitive Dysfunction OR Cognitive Dysfunctions OR Dysfunction, Cognitive OR Dysfunctions, Cognitive OR Cognitive Impairments OR Cognitive Impairment OR Impairment, Cognitive OR Impairments, Cognitive OR Cognitive Disorder OR Cognitive Disorders OR Disorder, Cognitive OR Disorders, Cognitive OR Mild Cognitive Impairment OR Cognitive Impairment, Mild OR Cognitive Impairments, Mild OR Impairment, Mild Cognitive OR Impairments, Mild Cognitive OR Mild Cognitive Impairments OR Cognitive Decline OR Cognitive Declines OR Decline, Cognitive OR Declines, Cognitive OR Mental Deterioration OR Deterioration, Mental OR Deteriorations, Mental OR Mental Deteriorations)** |
| **#2 (TS=(****Tai Chi OR Tai-ji OR Chi, Tai OR Tai Ji Quan OR Ji Quan, Tai OR Quan, Tai Ji OR Taiji OR Taijiquan OR T'ai Chi OR Tai Chi Chuan)** |
| #3 #1 AND #2 |
| #4 Baduanjin |
| #5 #1 AND #4 |
| #6 Wuqinxi |
| #7 #1 AND #6 |
| #8 Liuzijue |
| #9 #1 AND #8 |
| #10 Yijinjing |
| #11 #1 AND #10 |

| eTab. 2.e Search strategy for Sinomed database |
| --- |
| ("中国传统功法"[常用字段:智能] OR "功法"[常用字段:智能] OR "健身气功"[常用字段:智能] OR "八段锦"[常用字段:智能] OR "太极"[常用字段:智能] OR "太极拳"[常用字段:智能] OR "五禽戏"[常用字段:智能] OR "易筋经"[常用字段:智能] OR "六字诀"[常用字段:智能]) AND ("MCI"[常用字段:智能] OR "轻度认知障碍"[常用字段:智能] OR "轻度认知障碍疾病"[常用字段:智能] OR "认知障碍"[常用字段:智能]) |

| eTab. 2.f Search strategy for CNKI database |
| --- |
| 篇关摘： MCI + 轻度认知障碍 + 轻度认知障碍疾病 + 认知障碍  AND 篇关摘：中国传统功法 + 功法 + 健身气功 + 八段锦 + 太极 + 太极拳 + 五禽戏 + 易筋经 + 六字诀 |

| eTab. 2.g Search strategy for Wanfang database |
| --- |
| (题名或关键词：（ MCI or 轻度认知障碍 or 轻度认知障碍疾病)) and (题名或关键词：(中国传统功法 or 功法 or 健身气功 or 八段锦 or 太极 or 太极拳 or 五禽戏 or 易筋经 or 六字诀)) |

| eTab. 2.h Search strategy for VIP |
| --- |
| (题名或关键词：（ MCI or 轻度认知障碍 or 轻度认知障碍疾病 or 认知功能)) and (题名或关键词：(中国传统功法 or 功法 or 健身气功 or 八段锦 or 太极 or 太极拳 or 五禽戏 or 易筋经 or 六字诀)) |
